# Supplementary material for: Health Economic Aspects of Childhood Excess Weight: A Structured Review
Source: Children (Basel). 2022 Mar 24;9(4):461. doi: 10.3390/children9040461 (PMC9028108; doi:10.3390/children9040461)
Supplement: Supplementary file 1 [file children-09-00461-s001.zip › children-1600713-supplementary.pdf]

Table S1. Medline via Ovid search strategy and search outputs for studies on economic costs in childhood excess weight.

#	Searches	Results
1	exp Obesity/	218,633
2	Overweight/	25,790
3	obes*.mp.	371,839
4	exp Body Mass Index/	130,190
5	(bmi or adipos*).mp. [mp = title, abstract, original title, name of substance word, subject heading word, floating sub-heading word, keyword heading word, organism supplementary concept word, protocol supplementary concept word, rare disease supplementary concept word, unique identifier, synonyms]	299,231
6	1 or 2 or 3 or 4 or 5	597,405
7	exp Child/	1,938,185
8	(child* or school children or schoolchildren or pediatr* or paediatr* or boys or girls).mp.	2,619,724
9	7 or 8	2,619,724
10	exp "Costs and Cost Analysis"/	241,514
11	economic.ti,ab.	223,686
12	10 or 11	429,383
13	6 and 9 and 12	3061
14	exp animals/not humans/	4,775,603
15	13 not 14	3059
16	limit 15 to (english language and yr="2016 -Current")	1042

Table S2. Medline via Ovid search strategy combinations for studies on utilities in childhood excess weight.

Name of Strategy/Strategy Combination	Strategy
S1	"Health utilit*" OR "utilit* based" OR "utilit*-based" OR "utility* weight" OR "quality of life" OR QOL OR "health state valu*" OR "standard gamble" OR "time tradeoff" OR "time trade off" OR "time trade-off" OR "multi-attribute utilit*" OR CHU-9D OR CHU9D OR "child health utilit*" OR "short form 12" OR "short form 36" OR "EURoQoL 5D" OR "health utilit* index" OR HUI OR "quality of well being" OR "quality of well-being" OR "quality of wellbeing" OR QWB OR "assessment of quality of life 6D" OR AQoL-6D OR AQoL6D
S2	Child* OR adolescen* OR teen* OR youth OR pediatric OR paediatric
S3	"weight status" OR "body mass index" OR obes* OR BMI OR overweight OR adipos*
S4	"Cost-effectiv*" OR "cost effectiv*" OR "cost utilit*" OR "economic evaluation" OR "economic model*" OR "quality adjusted life year" OR QALY
S5	"discrete choice" OR "best worst scal*" OR "best-worst scal*" OR "multi attribute utilit*" OR "EQ-5D*" OR "EQ 5D*" OR "EQ5D" OR "Euroqol" OR "Euro qol" OR HUI2 OR "HUI 2" OR HUI3 OR "HUI 3" OR "short form survey-6D" OR "short form 6D" OR "SF 6D" OR SF6D OR "16D health related quality of life" OR "16D HRQOL" OR "17D health related quality of life" OR "17D HRQOL" OR "adolescent health utility measure" OR AHUM
S1 AND S2 AND S3	
S2 AND S3 AND S4	
S2 AND S3 AND S5	
	Searches conducted January 2021 (limit year = "2017–Current")

Table S3. Medline via Ovid search strategy and search outputs for studies on utilities in childhood excess weight.

#	Searches	Results
1	Health utilit*.mp.	2278
2	utilit* based.mp.	386
3	utilit*-based.mp.	386
4	utility* weight.mp.	40
5	exp Quality of Life/	202,778
6	QOL.mp.	41,029
7	health state valu*.mp.	433
8	standard gamble.mp.	861
9	time tradeoff.mp.	254
10	time trade off.mp.	1221
11	time trade-off.mp.	1221
12	multi-attribute utilit*.mp.	202
13	(CHU-9D or CHU9D).mp. [mp = title, abstract, original title, name of substance word, subject heading word, floating sub-heading word, keyword heading word, organism supplementary concept word, protocol supplementary concept word, rare disease supplementary concept word, unique identifier, synonyms]	66
14	child health utilit*.mp.	75
15	(short form 12 or short form 36 or EURoQoL 5D).mp. [mp = title, abstract, original title, name of substance word, subject heading word, floating sub-heading word, keyword heading word, organism supplementary concept word, protocol supplementary concept word, rare disease supplementary concept word, unique identifier, synonyms]	13,985
16	health utilit* index.mp.	940
17	HUI.mp.	1289
18	(quality of well being or quality of well-being or quality of wellbeing).mp. [mp = title, abstract, original title, name of substance word, subject heading word, floating sub-heading word, keyword heading word, organism supplementary concept word, protocol supplementary concept word, rare disease supplementary concept word, unique identifier, synonyms]	454
19	QWB.mp.	205
20	(assessment of quality of life 6D or AQoL-6D or AQoL6D).mp.	33
21	1 or 2 or 3 or 4 or 5 or 6 or 7 or 8 or 9 or 10 or 11 or 12 or 13 or 14 or 15 or 16 or 17 or 18 or 19 or 20	224,146
22	Child*.mp.	2,494,923
23	adolescen*.mp.	2,144,223
24	teen*.mp.	31,736
25	youth.mp. or Adolescent/	2,088,008
26	(pediatric or paediatric).mp. [mp = title, abstract, original title, name of substance word, subject heading word, floating sub-heading word, keyword heading word, organism supplementary concept word, protocol supplementary concept word, rare disease supplementary concept word, unique identifier, synonyms]	368,716
27	22 or 23 or 24 or 25 or 26	3,677,277
28	weight status.mp.	6287
29	body mass index.mp.	250,234
30	obes*.mp.	372,189
31	BMI.mp.	153,467
32	overweight.mp. or Overweight/	77,716
33	adipos*.mp.	161,116
34	28 or 29 or 30 or 31 or 32 or 33	661,534
35	Cost-effectiv*.mp.	143,070
36	cost effectiv*.mp.	143,070

37	cost utilit*.mp.	5119
38	economic evaluation.mp. or Cost-Benefit Analysis/	88,002
39	(economic model* or quality adjusted life year or QALY).mp.	14,791
40	35 or 36 or 37 or 38 or 39	191,039
41	(discrete choice or best worst scal* or best-worst scal* or multi attribute utilit*).mp.	2678
42	(EQ-5D* or EQ 5D* or EQ5D or Euroqol or Euro qol or HUI2 or HUI 2 or HUI3 or HUI 3).mp.	12,491
43	(short form survey-6D or short form 6D or SF 6D or SF6D or 16D health related quality of life or 16D HRQOL).mp.	890
44	(17D health related quality of life or 17D HRQOL or adolescent health utility measure or AHUM).mp.	4
45	41 or 42 or 43 or 44	15,419
46	21 and 27 and 34	2408
47	limit 46 to yr = "2017–Current"	732
48	27 and 34 and 40	898
49	limit 48 to yr = "2017–Current"	288
50	27 and 34 and 45	127
51	limit 50 to yr = "2017–Current"	46

46 => S1 AND S2 AND S3. 48 => S2 AND S3 AND S4. 50 => S2 AND S3 AND S5.

Table S4. Medline via Ovid search strategy and search outputs for studies on economic evaluation of childhood excess weight interventions.

#	Searches	Results
1	exp Obesity/	218,810
2	Obese.mp.	133,484
3	exp Overweight/	225,402
4	(BMI or body mass index).af.	298,964
5	Weight gain/	31,929
6	(Overweight or over weight or obesity or adipose).af.	480,218
7	exp Child/	1,939,453
8	exp Infant/	1,154,053
9	(Child* or adolescen* or infant*).af.	4,432,218
10	Schoolchild*.mp.	14,335
11	exp Adolescent/	206,1603
12	(Boys or girls or youth or youths).af.	212,956
13	(Teenage* or young person).af.	23,280
14	(Nutrition adj2 intervent*).af.	3851
15	((Obesity adj2 prevent*) or treat*).af.	6,246,518
16	Counsel?ing.mp.	124,432
17	exp support groups/	10,290
18	(Health Behaviour or Behaviour).mp.	213,262
19	exp Life Style/	96,462
20	exp Delivery of Health Care/	1,103,679
21	exp Social Support/	72,736
22	exp Family Practice/	65,410
23	exp Parent-Child Relations/	57,206
24	Food Habits.mp.	2151
25	exp Diet therapy/	55,529
26	exp Food Preferences/	14,714
27	exp Exercise therapy/	52,680
28	Physical activit*.mp.	120,934
29	Economic Evaluat*.mp.	13,208

#	Searches	Results
30	Cost*.ti.	125,266
31	Cost?Benefit*.mp.	95
32	Cost?Utilit*.mp.	11
33	Cost?Effective*.mp.	214
34	exp “costs and cost analysis”/	241,658
35	1 or 2 or 3 or 4 or 5 or 6	690,604
36	7 or 8 or 9 or 10 or 11 or 12 or 13	4,454,419
37	14 or 15 or 16 or 17 or 18 or 19 or 20 or 21 or 22 or 23 or 24 or 25 or 26 or 27 or 28	7,630,669
38	29 or 30 or 31 or 32 or 33 or 34	296,105
39	35 and 36 and 37 and 38	1015
40	limit 39 to yr = “2017–Current”	282

Table S5. Descriptive analysis of the key assessment items for individual economic evaluations.

First Author and Year of Publication	Country	Intervention(s) and Comparator(s)	Intervention Component(s) and Target(s) (Setting)	Type of Economic Evaluation (Study Perspective)	Target Population and Age Range	Time Horizon/Follow-Up for Trials	Currency Unit (Price Year)	Discount Rate(s)	Measure(s) of Consequence(s)	Results and Cost-Effectiveness	CHEERS Quality Score (%)
Goldfield, G. S., et al. 2001 [62]	United States	Mixed group treatment incorporating both group and individualised treatment versus group treatment only	PA, diet, and self-monitoring (Family-based)	Cost-effectiveness (Not stated/Provider perspective implied)	Families with obese 8- to 12-year-old children	1 year	U.S. dollars (not stated)	NA	Change in Z-BMI or change in percentage overweight	Both groups demonstrated equivalent weight control results; however, the mixed group was 2.8 times more expensive per family (USD 1390 vs. USD 491). The group treatment only was cost-effective	16.6/19 (87%)
Wang, L. Y., et al. 2003 [63]	United States	Planet Health, a school-based intervention targeting obesity versus 'no intervention'	PA, diet, and television viewing (School-based)	Cost-utility, cost-effectiveness, and cost-benefit analyses (Societal perspective)	Girls 10 to 14 years with mixed-weight status	25 years (age 40 to 65 years)	U.S. dollars (1996)	3% discount rate on future costs and benefits	Averted costs associated with cases of adulthood overweight prevented and QALY saved	An ICER of USD 4305 per QALY saved and a net saving of USD 7313 to society was estimated for the Planet Health programme relative to 'no intervention'. Cost-saving and cost-effective	21.5/23 (93%)
Brown, H. S., et al. 2007 [64]	United States	The CATCH[1] intervention versus control	PA, school curriculum/education, and modification to school food service (School-based with family- and home-based components)	Cost-utility and cost-benefit analyses (Societal perspective)	Children between the ages of 8 and 11 years across grades three, four, and five with mixed-weight status	25 years (age 40 to 65 years)	U.S. dollars (2004)	3% discount rate on future costs and benefits	QALY saved and averted medical and labour productivity costs	An ICER of USD 900 per QALY saved and a net benefit of USD 68,125 to society was estimated for the CATCH programme relative to control. Cost-effective	22.5/24 (94%)
Moodie, M., et al. 2008 [85]	Australia	A behavioural intervention involving GP consultations at home (the LEAP trial) versus 'no intervention' control	Lifestyle, diet, PA, and GP consultations (Family-based and GP-mediated)	Cost-effectiveness and cost-utility analyses (Societal perspective)	Children aged 5 to 9 years with a BMI z-score of ≥ 3.0 (overweight and moderately obese)	Lifetime	Australian dollars (2001)	3% discount rate on future costs and benefits	DALY saved	Compared to the control, the intervention resulted in a net cost per DALY saved of AUD 4670. Cost-effective	21.5/22 (98%)
Peterson, M., et al. 2008 [65]	United States	State-wide social marketing: (exposure to varying levels of	PA, television, and billboard advertising	Cost-effectiveness analysis (Societal perspective)	Children 12 to 17 years with mixed-weight	NA	U.S. dollars (not stated)	NA	Increase in PA (measure not specified)	Cost per person to become more active was lowest for billboards alone at USD 5.11	9.5/17 (56%)

First Author and Year of Publication	Country	Intervention(s) and Comparator(s)	Intervention Component(s) and Target(s) (Setting)	Type of Economic Evaluation (Study Perspective)	Target Population and Age Range	Time Horizon/Follow-Up for Trials	Currency Unit (Price Year)	Discount Rate(s)	Measure(s) of Consequence(s)	Results and Cost-Effectiveness	CHEERS Quality Score (%)
		advertisements) versus No advertisements	(Population-based)		status attending public schools					but cost USD 8.87 per person for the entire campaign. Billboards were potentially more cost-effective than television advertisements in high traffic areas	
Wake, M., et al. 2008 [86]	Australia	A behavioural intervention involving GP consultations at home (the LEAP trial) versus 'no intervention' control	Lifestyle, diet, PA, and GP consultations (Family-based and GP-mediated)	Cost-consequence analysis (Societal perspective)	Children aged 5 to 9 years with a BMI z-score of ≥ 3.0 (overweight and moderately obese)	15 months after randomisation	Australian dollars (2003)	NA	Children's change in BMI, PA, and dietary habits	Mean change in BMI z-score not statistically significant at 2 and 1.92 for the intervention and control, respectively. Daily dietary habits improved significantly in the intervention. The additional cost from the intervention was AUD 4094. Not cost-effective	17/19 (89%)
Wang, L. Y., et al. 2008 [66]	United States	An after-school intervention (MCG FitKid) versus usual after-school care control	Diet, PA, and academic enrichment activities (School-based)	Cost-effectiveness analysis (Societal perspective)	Third-grade children with mean age of 8.7 years with mixed-weight status	1 year after randomisation	U.S. dollars (2003)	NA	Reduction in percent body fat (%BF)	The study found that students who attended at least 40% of the intervention reduced %BF by 0.76% at an additional cost of USD 317/student, compared with students in the control group. Potentially cost-effective	18/19 (95%)
Janicke, D. M., et al. 2009 [67]	United States	Parent-only intervention versus family-based intervention	Diet and PA (Rural community setting)	Cost-effectiveness analysis (Societal perspective)	Overweight or obese children aged 8 to 14 years and their parent(s)	10-month follow-up after randomisation	U.S. dollars (not stated)	NA	BMI unit reduction	The total cost per child for the parent-only and family interventions were USD 521 and USD 872, respectively. No significant difference in weight status change at follow-up. Parent-only potentially cost-effective	15/20 (75%)
Kalavainen, M., et al. 2009 [121]	Finland	Family-based group treatment (15 separate sessions for parents and children) versus	Diet and PA (Primary health care)	Cost-effectiveness analysis (Societal perspective)	Obese children 7 to 9 years old	12 months post-baseline	Euros (2004)	NA	BMI unit reduction	The additional costs for a 0.1 reduction in BMI z-score for the family-based group treatment were EUR 266 compared with routine	18/20 (90%)

First Author and Year of Publication	Country	Intervention(s) and Comparator(s)	Intervention Component(s) and Target(s) (Setting)	Type of Economic Evaluation (Study Perspective)	Target Population and Age Range	Time Horizon/Follow-Up for Trials	Currency Unit (Price Year)	Discount Rate(s)	Measure(s) of Consequence(s)	Results and Cost-Effectiveness	CHEERS Quality Score (%)
		routine counselling (two appointments for children)								counselling. Inconclusive: family-based group treatment more effective but also costlier	
Magnus, A., et al. 2009 [87]	Australia	A ban on television advertisements for EDNP foods and beverages versus current practice	Diet and legislation (Population-based, home-setting)	Cost-effectiveness and cost-utility analyses (Societal perspective)	All children aged 5 to 14 years	Lifetime	Australian dollars (2001)	3% discount rate on future costs and benefits	Changes in BMI and DALYs saved	The intervention had a gross ICER of AUD 3.70 per DALY saved and cost per BMI unit saved of AUD 0.33. Cost-effective	22/22 (100%)
Moodie, M., et al. 2009 [88]	Australia	WSB programme versus 'current practice' defined as 'do nothing'	PA: increase the number of children walking to school (Population-based, school-based)	Cost-effectiveness and cost-utility analyses (Societal perspective)	Primary school children aged 5 to 7 years with mean BMI (Kg/m2) 16.24, 16.47 for boys and girls, respectively	Lifetime	Australian dollars (2001)	3% discount rate on future costs and benefits	BMI unit saved and DALY saved	The ICER for the WSB intervention was estimated at a net cost per DALY saved of AUD 0.76 million but the evidence base underpinning the results was deemed weak by the authors. Not effective or cost-effective	22/22 (100%)
McAuley, K. A., et al. 2010 [111]	New Zealand	The APPLE project: the provision of community activity coordinators at each intervention school versus usual care	Diet, PA, and lifestyle-based (School and Community-based)	Cost-effectiveness and cost-utility analyses (Societal perspective)	Children aged 5 to 12 years representative of the wider population in this age group	4 years	New Zealand dollars (2006)	5% discount rate per annum for costs	Reduction in weight z-score and QALY gain (HUI values)	The implementation cost for the intervention was NZD 664–1708 per kg of weight gain prevented over 4 years with weight z-score reduction was 0.17 units relative to control, but mean utility values did not differ between the groups. Potentially cost-effective	20.5/22 (93%)
Moodie, M. L., et al. 2010 [89]	Australia	The AASC programme: 2 to 3 sessions per week of PA programmes during the after-school period versus no intervention	PA: after school period (Population-based, school-based)	Cost-effectiveness and cost-utility analyses (Societal perspective)	Children aged 5 to 11 years (grade 4 to 6) of mixed weight status	Lifetime	Australian dollars (2001)	3% discount rate on future costs and benefits	BMI unit saved and DALY saved	The ICER for the intervention was estimated at a net cost per DALY saved of AUD 82,000. Not cost-effective	22/22 (100%)
Moodie, M., et al. 2011 [90]	Australia	TravelSMART Schools Curriculum programme versus current practice	PA (Population-based, school-based)	Cost-effectiveness and cost-utility analyses (Societal perspective)	Children aged 10 to 11 years of mixed weight status	Lifetime	Australian dollars (2001)	3% discount rate on future costs and benefits	BMI unit saved and DALY saved	The ICER for the intervention was estimated at a net cost per DALY saved of AUD 117,000. Not cost-effective	22/22 (100%)

First Author and Year of Publication	Country	Intervention(s) and Comparator(s)	Intervention Component(s) and Target(s) (Setting)	Type of Economic Evaluation (Study Perspective)	Target Population and Age Range	Time Horizon/Follow-Up for Trials	Currency Unit (Price Year)	Discount Rate(s)	Measure(s) of Consequence(s)	Results and Cost-Effectiveness	CHEERS Quality Score (%)
Martínez, P. M., et al. 2011 [115]	Spain	Three 90-minutes sessions per week of recreational PA conducted after school hours on school premises versus usual afterschool care	PA (School-based)	Cost-consequence analysis (Societal perspective)	Children aged 9 to 10 years of mixed weight status	7 months from baseline	Euros (2005)	NA	Decrease in triceps skinfold thickness and decrease in the percentage of body fat	The intervention costs EUR 269.83/year/child while the usual afterschool care was estimated at EUR 844.56/year/child. The decrease in the percentage of body fat for the intervention group ranged from −0.59% to −0.67%. Potentially cost-effective	17/20 (85%)
Wang, L. Y., et al. 2011 [68]	United States	Planet Health, a school-based intervention targeting obesity and DWCB versus 'no intervention'	PA, diet, DWCB prevention (purging or diet pill use), and television viewing (School-based)	Cost-utility analysis (Societal perspective)	Girls 10 to 14 years with mixed-weight status	10 years	U.S. dollars (2010)	3% discount rate on future costs and benefits	QALY gained (15D instrument)	With a net savings of USD 14,238 and a gain of 4.8 QALYs, the ICER for the intervention was estimated at USUSD 2966 per QALY gained. Potentially cost-effective	22/22 (100%)
Hollingworth, W., et al. 2012 [100]	United Kingdom	Lifestyle interventions (identified in literature review of 10 RCTs) to treat overweight and obese children versus no intervention	Diet and PA (Hospital and community-based)	Cost-effectiveness analysis (UK National Health Service perspective)	Hypothetical cohorts of overweight or obese children aged 4 to 5 and 10 to 11 years in state schools	Lifetime	GBP (not stated)	3.5% discount rate on future costs and health outcomes	Life year gained	For children aged 10 to 11, the discounted cost per LYG was estimated at GBP 13,589. Similar results reported for children aged 4 to 5 years. Lifestyle interventions potentially cost-effective	21/23 (91%)
Kesztyüs, D., et al. 2013 [113]	Germany	The URMEL-ICE intervention integrated into the usual school curriculum consisting of teaching, activity breaks and homework assignments versus usual care	Diet, PA, and media (School-based)	Cost-effectiveness analysis (Societal perspective)	Primary school children (second graders), mixed-weight group, mean ages 7.64 and 7.53 in the intervention and control groups, respectively	1 year	Euros (2008)	NA	WC gain prevented, WHtR gain prevented, and BMI difference between groups	The ICER was EUR 11.11 per cm WC and EUR 18.55 per unit WHtR gain prevented for the intervention group with respect to the control group. Assuming a maximum WTP of EUR 35, cost-effective	18/19 (95%)

First Author and Year of Publication	Country	Intervention(s) and Comparator(s)	Intervention Component(s) and Target(s) (Setting)	Type of Economic Evaluation (Study Perspective)	Target Population and Age Range	Time Horizon/Follow-Up for Trials	Currency Unit (Price Year)	Discount Rate(s)	Measure(s) of Consequence(s)	Results and Cost-Effectiveness	CHEERS Quality Score (%)
Meng, L., et al. 2013 [60]	China	Four groups: nutrition education; PA; nutrition education and PA (comprehensive intervention); and control (usual care)	Diet and PA (School-based)	Cost-effectiveness analysis (Societal perspective)	Children 6 to 13 years of mixed-weight status	1 year from baseline	CNY/U.S. dollars (2010)	NA	BMI z-score avoided, overweight and obesity case avoided	The comprehensive intervention had an ICER of USD 120.3 per 1 kg/m2 BMI reduction, USD 249.3 per BMI z-score, and USD 1308.9 per one overweight and obesity case avoided compared with the control group. Comprehensive intervention cost-effective	17.5/23 (76%)
Moodie, M. L., et al. 2013 [91]	Australia	BAEW: an intervention to increase PA and healthy eating versus usual practice	Reduction of television viewing, diet, and PA (Community-based, primary school setting)	Cost-effectiveness and cost-utility analyses (Societal perspective)	Children aged 4 to 12 years mixed-weight status	Lifetime	Australian dollars (2006)	3% discount rate on future costs and health outcomes	BMI units saved and DALYs averted	The BAEW yielded a net cost per DALY saved of AUD 29,798 in comparison with the comparator. Cost-effective	22/22 (100%)
Epstein, L. H., et al. 2014 [69]	United States	Two assumptions of P&C treated separately: P&C-1 (P&C treated on separate days) and P&C-2 (P&C treated on same days) versus FBT obesity treatment.	Diet and PA (Medical school setting)	Cost-effectiveness analysis (Societal perspective)	Children 8 to 12 years old, at or above the 85th BMI percentile, and had an overweight/obese parent	1 year from baseline	U.S. dollars (not stated)	NA	Unit of weight loss (defined in children as % over BMI)	ICERs were not estimated: the costs per unit weight lost for children were USD 209.17 per % over BMI, USD 1036.50 per % over BMI and USD 973.98 per % over BMI for FBT PC-1 and PC-2 groups, respectively. FBT potentially cost-effective	16.5/19 (87%)
Hayes, A., et al. 2014 [92]	Australia	Home-based early intervention: eight home visits by specially trained community nurses (including one visit at 30–36 weeks gestational age) versus usual care	Diet and PA (Home-based)	Cost-effectiveness analysis (Health care funder perspective)	Infants followed and received the intervention for the first 2 years of life	2 years from baseline	Australian dollars (2012)	5% discount rate on future costs and health outcomes	Reduction in BMI z-score/unit BMI avoided	The incremental cost-effectiveness ratio was AUD 4230 per unit BMI avoided and AUD 631 per 0.1 reduction in BMI z-score or AUD 2697 per unit BMI avoided and AUD 376 per 0.1 reduction in BMI z-score, if the intervention cost per child was AUD 1309 or AUD 709, respectively. Potentially cost-effective	20/20 (100%)

First Author and Year of Publication	Country	Intervention(s) and Comparator(s)	Intervention Component(s) and Target(s) (Setting)	Type of Economic Evaluation (Study Perspective)	Target Population and Age Range	Time Horizon/Follow-Up for Trials	Currency Unit (Price Year)	Discount Rate(s)	Measure(s) of Consequence(s)	Results and Cost-Effectiveness	CHEERS Quality Score (%)
Hollinghurst, S., et al. 2014 [101]	United Kingdom	Primary care clinic versus COCO hospital clinic and Mandometer® training versus COCO hospital clinic	Diet and PA (Primary care, home and hospital-based)	Cost-effectiveness analysis (Health care funder perspective)	Children aged between 5 and 16 with BMI ≥98th centile (the PC-COCO study); children and young people aged 9 to 17 years with a BMI SDS >95th centile (the Mandometer® study)	1 year from baseline	GBP (not stated)	NA	Reduction in BMI z-score	The ICER for a primary care clinic compared to COCO hospital clinic was GBP 175 per 0.1-point improvement in BMI SDS while the ICER for Mandometer® training compared to hospital clinic was GBP 574 per 0.1-point improvement in BMI SDS. Mandometer® training potentially cost-effective	17.5/20 (88%)
Rush, E., et al. 2014 [110]	New Zealand	Project Energise, a multicomponent through-school PA and nutrition programme versus usual care (unenergised)	Diet and PA (School-based)	Cost-utility analysis (Health care funder perspective)	Younger (6 to 8 years of age) and older primary school children (9 to 11 years of age) projected nationally/Maori population	Lifetime	New Zealand dollars (2011)	3.5% discount rate on future costs and health outcomes	QALY gained	Compared to usual care, the ICER was NZD 30,438 for the younger and NZD 24,690 for the older children, and lower for Maori (younger NZD 28,241, older NZD 22,151) and for the middle socioeconomic status schools (NZD 23,211, NZD 17,891) per QALY gained. Potentially cost-effective	22/24 (92%)
Barrett, J. L., et al. 2015 [70]	United States	The 'Active PE' policy: a requirement that 50% of PE time of state's elementary school PE curriculum be devoted to MVPA versus current practice	PA (School-based)	Cost-effectiveness analysis (Societal perspective)	Children aged 6 to 11 years	10 years	US dollars (2014)	3% discount rate on future costs and health outcomes	BMI units reduced	The intervention was estimated to cost USD 1720 per BMI unit reduced over the modelled 10-year period. This translated to an estimated USD 60.5 million in health care costs averted. Potentially cost-effective	22.5/23 (98%)
Long, M. W., et al. 2015 [71]	United States	Implementing a sugar-sweetened beverage excise tax	Diet and tax (Multiple settings	Cost-utility, cost-effectiveness, cost-benefit analyses	U.S. population aged ≥2 years at baseline	2 years (10 years for the	U.S. dollars (2014)	3% discount rate on future costs and	BMI units reduced	Over 2 years, the cost per BMI unit reduced due to the tax was estimated at USD 8.54 in	23/23 (100%)

First Author and Year of Publication	Country	Intervention(s) and Comparator(s)	Intervention Component(s) and Target(s) (Setting)	Type of Economic Evaluation (Study Perspective)	Target Population and Age Range	Time Horizon/Follow-Up for Trials	Currency Unit (Price Year)	Discount Rate(s)	Measure(s) of Consequence(s)	Results and Cost-Effectiveness	CHEERS Quality Score (%)
		versus the current practice (SSB)	possible, population-based)	(Societal perspective)		general population)		health outcomes		children and youth aged 2 to 19 years of age. Potentially cost-effective	
Sonneville, K. R., et al. 2015 [72]	United States	Eliminating tax subsidy of TV advertising to children (TV AD) versus current practice	Diet, television viewing, and tax (Home-based)	Cost-utility, cost-effectiveness, cost-benefit analyses (Societal perspective)	Children aged 2 to 19 years	10 years	U.S. dollars (2014)	3% discount rate on future costs and health outcomes	Net cost saved per dollar spent and QALY gained	The net reduction in cost and the total QALYs gained over 10 years were USD 343 million and 4540, respectively. The net cost savings per dollar spent was estimated at USD 38. Potentially cost saving	23/23 (100%)
Wright, D. R., et al. 2015 [73]	United States	Early care and education policy change (ECE) versus current practice	PA, diet, and television viewing (Multiple settings possible, including childcare setting)	Cost-utility, cost-effectiveness, cost-benefit analyses (Societal perspective)	Children aged 3 to 5 years	11 years	U.S. dollars (2014)	3% discount rate on future costs and health outcomes	BMI units reduced and net cost saved per dollar spent	The ICER for the SSB was estimated at USD 57.80 per BMI unit avoided over two years and a net health care cost savings of USD 51.6 was estimated for a 10-year time horizon. Potentially cost-effective	22/22 (100%)
Gortmaker, S. L., et al. 2015 [74]	United States	Four childhood obesity interventions: SSB excise tax; eliminating tax subsidy of TV AD to children; ECE policy change; and active PE.	PA, diet, television viewing, tax, and education (Multiple settings possible)	Cost-utility, cost-effectiveness, cost-benefit analyses (Societal perspective)	Children aged SSB 2 to 19 years; TV AD 2 to 19 years; ECE 3 to 5 years; and Active PE 6 to 11 years.	10 years	U.S. dollars (2014)	3% discount rate on future costs and health outcomes	BMI units reduced and net cost saved per dollar spent (DALYs averted/QALYs gained not fully reported)	The net cost saved per dollar spent was USD 55, USD 38, and USD 6 for SSB, TV AD, and ECE, respectively. All four preventive interventions potentially more cost-effective clinical interventions	22.5/23 (98%)
Sutherland, R., et al. 2016 [93]	Australia	The PA4E1 multi-component intervention versus usual care	Diet and PA (School-based, low-income communities)	Cost-effectiveness analysis (Societal perspective)	Children in their first year of high school, mean age 12 years old of mixed weight status groups	2 years	Australian dollars (2014)	Not stated	Unit (10%) reduction in BMI z-score, mean minute of MVPA gained and MET minute gained	The ICERs were AUD 56 per additional minute of MVPA/day, AUD 749 per MET hours gained per person/day and AUD 563 per 10% reduction in BMI z-score. Potentially cost-effective	18.5/20 (93%)

First Author and Year of Publication	Country	Intervention(s) and Comparator(s)	Intervention Component(s) and Target(s) (Setting)	Type of Economic Evaluation (Study Perspective)	Target Population and Age Range	Time Horizon/Follow-Up for Trials	Currency Unit (Price Year)	Discount Rate(s)	Measure(s) of Consequence(s)	Results and Cost-Effectiveness	CHEERS Quality Score (%)
Graziose, M. M., et al. 2017 [75]	United States	FHC, an obesity prevention nutrition education curriculum delivered over 1 year versus current practice (no-intervention)	Diet, PA, and screen time (School-based)	Cost-utility analysis (Societal perspective)	All New York City fifth-grade public school students (mean age 10 years)	Lifetime	U.S. dollars (2012)	3% discount rate on future costs and health outcomes	QALY saved	An ICER of USD 275 per QALY saved was estimated for the intervention. Cost-effective	23.5/24 (98%)
Robertson, W., et al. 2017 [102]	United Kingdom	'Families for Health', a 10-week family-based childhood obesity treatment intervention versus usual care	Diet, PA, parenting skills, and relationship skills (Community-based)	Cost-utility analysis (NHS and Personal Social Services perspective)	Children aged 6 to 11 years who were overweight or obese and their parents or carers	1-year post-randomisation	GBP (2013/14)	NA	Change in children's BMI z-score and QALYs gained (EQ-5D-Y)	The ICER for the Families for Health programme was estimated at GBP 552,175 per QALY gained. Not cost-effective	21/21 (100%)
Klebanoff M. J., et al. 2017 [76]	United States	Bariatric surgery (either gastric bypass or sleeve gastrectomy) versus no surgery	Bariatric surgery (Hospital setting)	Cost-utility analysis (Health care provider)	Adolescents (mean age 17 years) with severe obesity	3 years base case	U.S. dollars (2015)	3% annual rate for cost and QALYs	QALY gained (Instrument not stated)	ICER using a WTP threshold of USD 100,000 per QALY gained: at 3 years USD 154,684 per QALY (surgery dominated); at 5 years USD 91,032 per QALY, surgery potentially cost-effective	18/24 (75%)
Cradock, A. L., et al. 2017 [77]	United States	Six prevention strategies: (Active PE; Active Recess; Active School Day; Healthy Afterschool; New Afterschool Programmes; and Hip Hop to Health, Jr.) versus the status quo	PA: state-wide and national implementation (School, afterschool, and childcare settings)	Cost-effectiveness and cost-benefit analyses (Societal perspective)	Children aged 3 to 14 regardless of BMI status	10 years (2015 to 2025)	U.S. dollars (2014)	3% annual rate for cost	BMI unit reduction, cases of childhood obesity prevented, and health care cost savings.	All interventions showed a potential to prevent cases of obesity. The ICER for New Afterschool Programme was dominant, while the ICERs for the remaining interventions ranged from USD 361 to USD 2825 per BMI unit change per person. All interventions potentially cost-effective	21.5/24 (90%)
Ekwaru, J. P., et al. 2017 [117]	Canada	The Alberta Project Promoting Active Living and healthy Eating in Schools (APPLE) Schools (the intervention	PA and diet (Elementary schools)	Cost-utility analysis (Societal perspective)	Grade five students (about 10 years of age); weight status included normal weight,	Lifetime	Canadian dollars (2008)	3% annual rate for cost and QALYs	QALY gained (Instrument not stated)	At a cost-effectiveness threshold of CAD 50,000, CAD 33,421 per QALY gained.	22/24 (92%)

First Author and Year of Publication	Country	Intervention(s) and Comparator(s)	Intervention Component(s) and Target(s) (Setting)	Type of Economic Evaluation (Study Perspective)	Target Population and Age Range	Time Horizon/Follow-Up for Trials	Currency Unit (Price Year)	Discount Rate(s)	Measure(s) of Consequence(s)	Results and Cost-Effectiveness	CHEERS Quality Score (%)
		programme) versus general schools (No intervention)			overweight, and obesity						
Larsen, K. T., et al. 2017 [118]	Denmark	Camp group (CG), a six-week high-intensity day-camp intervention versus Standard group (SG), a six-week low-intensity intervention.	PA: motivation-enhancing PA and health education (Municipal setting)	Cost-effectiveness analysis (Societal perspective)	Overweight and obese fifth-grade children with a mean age of 12 years	Two years follow-up	DKK (2012)	Not stated	Changes in BMI and BMI z-score	The ICER was DDK 149,669 per unit decrease in BMI z-score for CG compared with SG in the base case scenario. Insufficient information/potentially cost-effective	16.5/24 (69%)
Lee, B. Y., et al. 2017 [78]	United States	Two levels of PA: 25 min of high-calorie-burning PA three times a week, for children 6 years and older; and 60 minutes of moderate PA each day, for children ages 6–17 years.	PA (School/community)	Cost-consequences analysis (Societal perspective)	A simulation sample representative of the U.S. population of children (6 to 17 years) with normal, overweight, and obese status	Lifetime	U.S. dollars (2016)	3% for costs	Lifetime medical care costs, lost productivity, reduction in cases of obesity, quality-adjusted life-years (QALY instrument not stated)	Increasing the current level of children who get 25 minutes of high-calorie-burning PA from 31.9% to 75% was estimated to save a total of 18,913,447 QALYs. At 31.9%, an annual net present value of USD 1.1 trillion in direct medical costs was estimated. Potentially cost-effective	19.5/24 (81%)
Makkes, S., et al. 2017 [107]	The Netherlands	Two intensive 1-year lifestyle treatment interventions consisting of either an inpatient period of 2 months (short-stay group) or 6 months (long-stay group).	PA (Clinical setting: specialised childhood obesity centre)	Cost-effectiveness and cost-utility analyses	8 to 19 years with severe obesity	1 year of treatment and 1 year of follow-up	Euros (2010)	4% and 1.5% for costs and outcomes respectively	BMI z-scores and QALYs (EQ5D)	The difference in BMI z-score and QALYs between both groups after 24 months was very small and possibly statistically insignificant. The ICER was one point higher in SDS-BMI per savings of EUR 1,479,463 for the short-stay compared with the long-stay group. Short-stay cost-effective	20/24 (83%)
Quattrin, T., et al. 2017 [79]	United States	Family-based behavioural treatment (FBT) and an attention-controlled	PA and diet: parent and child-focused (Clinical setting: patient-	Cost-effectiveness analysis (Societal perspective)	Children 2 to 5 years of age with overweight and obesity (BMI over the 85th	1 year of treatment and 1 year of follow-up	U.S. dollars (2013)	NA	Child percent over BMI (%OBMI) change and parent BMI change	In the intention-to-treat group, the FBT ICER compared to IC was USD 116.1 per unit reduction in %OBMI for children and parents, the ICER	19.5/24 (81%)

First Author and Year of Publication	Country	Intervention(s) and Comparator(s)	Intervention Component(s) and Target(s) (Setting)	Type of Economic Evaluation (Study Perspective)	Target Population and Age Range	Time Horizon/Follow-Up for Trials	Currency Unit (Price Year)	Discount Rate(s)	Measure(s) of Consequence(s)	Results and Cost-Effectiveness	CHEERS Quality Score (%)
		information control (IC) group	centred medical home)		percentile for their age and sex) with their parents (BMI ≥ 25)					was USD 353.8 per kilogram reduction in weight. FBT potentially cost-effective	
Beets, M. W., et al. 2018 [80]	United States	Two afterschool programs (ASPs): immediate or delayed	PA and diet (Schools, community/recreation, and faith locations)	Cost-consequences analysis	Children aged 5 to 12 years including normal weight, overweight, and obese weight status	End of the 2-year trial	U.S. dollars (2015)	NA	Changes in MVPA and changes in number of days fruits/vegetables, water, desserts, and SSBs were served.	In immediate/delayed groups, an increase of USD 0.05/USD 0.26 and USD 0.23/USD 0.08 per child per weekly enrolment fee was required to increase % of boys and girls accumulating 30 min or more of MVPA by 1%, respectively. Immediate potentially cost-effective in boys but not in girls	17.5/24 (73%)
An, R., et al. 2018 [81]	United States	Placing water dispensers at school cafeterias nationwide to promote lunchtime plain water consumption versus no action	Drinking water (School-based)	Cost-benefit analysis (Societal perspective)	All school children in primary, secondary, and high schools	Lifetime	U.S. dollars (2016)	3% discount rate on future costs and benefits	Number of cases of overweight/obesity prevented expressed in terms of per capita annual medical cost of adult overweight/obesity	The net benefit of the intervention was USD 174 per student. A net benefit of USD 199 and USD 149 per student was estimated for boys and girls, respectively. Potentially cost-effective	18.5/24 (77%)
Anderson, Y. C., et al. 2018 [112]	New Zealand	Two home-based multi-disciplinary child obesity intervention programmes (high-intensity and low-intensity) versus conventional hospital-based care	Multi-disciplinary assessment and advice to promote healthy diet and PA choices (Home-based, community-based, and hospital-based care)	Cost-effectiveness analysis (Health funder perspective)	Children aged 5–16 years with a body mass index (BMI) ≥98th centile or BMI >91st centile with weight-related comorbidities. In the comparator arm, children	12 months from baseline	New Zealand dollars (2016)	NA	BMI z-score	The low-intensity group was NZD 939 lower than the conventional group. In the high-intensity intervention group was NZD 155. Both low- and high-intensity groups had mean BMI z-score reductions of 0.03 more than the conventional group. Potentially cost-effective	18.5/24 (77%)

First Author and Year of Publication	Country	Intervention(s) and Comparator(s)	Intervention Component(s) and Target(s) (Setting)	Type of Economic Evaluation (Study Perspective)	Target Population and Age Range	Time Horizon/Follow-Up for Trials	Currency Unit (Price Year)	Discount Rate(s)	Measure(s) of Consequence(s)	Results and Cost-Effectiveness	CHEERS Quality Score (%)
					were aged 4–15 years.						
Brown, V., et al. 2018 [94]	Australia	Legislation to restrict TV advertising of food and beverages high in fat, sugar, and salt (HFSS) until 9:30 p.m. versus baseline or usual viewing	Diet and legislation (Population-based, home-based)	Cost-effectiveness analysis (Societal perspective)	All children 5–15 years of age	Lifetime	Australian dollars (2010)	3% discount rate on future costs and health benefits	Reductions in energy intake (mean KJ/day), reductions in BMI and HALY saved	The intervention was dominant with 88,396 HALYs saved and a total cost savings of AUD777.9M at the population level over the lifetime. Potentially cost-effective	23/24 (96%)
Conesa, M., et al. 2018 [116]	Catalonia, Spain	Educació en Alimentació Programme (EdAl): a set of educational interventions focused on improving lifestyle choices versus control (usual care)	Diet and PA (School-based)	Cost-effectiveness analysis (Societal perspective)	Children aged 7–8 years, comprising underweight to obese weight status but about 70% being normal weight in both arms	End of the trial: 28 months (2.3 years)	Euros (2007)	NA	Number of obesity cases avoided, the decrease in obesity prevalence, the decrease in BMI units, and the decrease in BMI z-score units	Cost-effectiveness analysis not performed for girls as the intervention was not effective. For boys, the ICERs (intervention relative to control) were 968.66 EUR/case of obesity avoided, 44.68 EUR/BMI one-unit decrease, 3.56 EUR/1% obesity prevalence reduction and 65.16 EUR/BMI z-score one-unit decrease. Cost-effective in boys	17.5/24 (73%)
Döring, N., et al. 2018 [119]	Sweden	PRIMROSE intervention: an early childhood obesity intervention delivered in the first 4 years of life versus usual care	Diet and PA; Motivational Interviewing (Clinical; child health care centres)	Cost-effectiveness analysis (Societal perspective)	Pre-school children aged 9 months of age of mixed weight status	End of the trial: child at age 4 (48 months)	Euros (2015)	Not stated	BMI unit prevented	In the base case analysis, the ICER was EUR 3109 per 1 BMI unit prevented. Inconclusive due to high uncertainty around effect measure	17/24 (71%)
Panca, M., et al. 2018 [103]	Greater London, England	The Healthy Eating Lifestyle Programme (HELP) compared with enhanced standard care	Diet and PA: motivational multicomponent lifestyle-modification intervention	Cost-utility analysis (U.K. National Health Service)	Young people with obesity aged 12 to 18 years	One-year time horizon (treatment ended at 6 months and follow-up	GBP (2013/14)	NA	QALY gained (EQ-5D-3L)	The ICER of the HELP versus enhanced standard care was GBP 120,630 per QALY gained and there were no differences in adjusted QALYs between	21.5/24 (90%)

First Author and Year of Publication	Country	Intervention(s) and Comparator(s)	Intervention Component(s) and Target(s) (Setting)	Type of Economic Evaluation (Study Perspective)	Target Population and Age Range	Time Horizon/Follow-Up for Trials	Currency Unit (Price Year)	Discount Rate(s)	Measure(s) of Consequence(s)	Results and Cost-Effectiveness	CHEERS Quality Score (%)
			(Community setting)			for another 6 months)				both groups. HELP not cost-effective	
Reilly, K. L., et al. 2018 [95]	Australia	Three multicomponent interventions (high, medium, and low intensity) to enhance implementation of a healthy canteen policy versus usual support	Diet-based (School-based)	Cost-effectiveness analysis (Health service delivery perspective)	Students 5 to 12 years old in primary schools of mixed weight status	End of trials (12 months). The Medium trial was scaled from 9 months to 12 months	Australian dollars (2015)	NA	Percentage point increase in the proportion of schools that adhered to the policy	The ICERs for the three interventions were AUD 2982 (high intensity), AUD 2627 (medium intensity) and AUD 4730 (low intensity) per percent increase in the proportion of schools reporting 'adherence' compared to usual support. Medium intensity potentially cost-effective	20.5/24 (85%)
Vidmar, A. P., et al. 2019 [82]	United States	iPhone® app intervention with clinic visits, targeting addictive eating behaviour versus EMPOWER clinic intervention consisting of a team of health care professionals who evaluate patients	App-based (Mobile health technologies) and clinical evaluation-based (Clinic and home-based)	Cost-consequences analysis (Provider perspective)	Obese adolescents 12 to 18 years without significant obesity comorbidities but exhibited signs of addictive eating	6 months post-baseline	U.S. dollars (not stated)	NA	Mean change in BMI z-score and excess percent over the 95th percentile (%BMIp95)	App participants had lower total costs per patient (USD 855.15 vs. USD 1428.00) than the EMPOWER clinic participants. Both app participants and EMPOWER completers demonstrated comparable decreases in BMI z-scores and %BMIp95 6 months after baseline. App intervention potentially cost-effective	16/24 (67%)
Amies-Cull, B., et al. 2019 [104]	England	20% reduction in the sugar content of certain high sugar products versus no reduction	Diet-based (Population-based)	Cost-consequences analysis (NHS England perspective)	The general population aged 4 to 80 years, specifically capturing children age group cohorts of 4 to 10 years and 11 to 18 years.	10 years in the base case	GBP (not stated)	Not stated	Calorie change, weight change, and BMI change for children and adults. QALYs for adults only.	A potential reduction in obesity among 4–10-year-olds by 5.5%, 11–18-year-olds by 2.2% was estimated for the intervention. Potentially cost-effective	19.5/24 (82%)

First Author and Year of Publication	Country	Intervention(s) and Comparator(s)	Intervention Component(s) and Target(s) (Setting)	Type of Economic Evaluation (Study Perspective)	Target Population and Age Range	Time Horizon/Follow-Up for Trials	Currency Unit (Price Year)	Discount Rate(s)	Measure(s) of Consequence(s)	Results and Cost-Effectiveness	CHEERS Quality Score (%)
Ananthapavan, J., et al. 2019 [96]	Australia	Hypothetical community-based obesity prevention interventions (CBIs) versus control	Diet, PA, change in school infrastructure, and changes to the obesogenic environment within the community (Community-based)	Cost-effectiveness analysis (Societal perspective)	Australian children aged 5 to 18 years	Lifetime	Australian dollars (2010)	3% discount rate on future costs and benefits	Change in BMI and HALY gained	For CBIs, the ICER was AUD8155 per HALY gained at a willingness to pay threshold of AUD 50,000 per HALY. Cost-effective	23.5/24 (98%)
Brown, V., et al. 2019 [97]	Australia	Seven hypothetical scenarios of varying effect (reduction in BMI z-score) maintenance and subsequent decay to no effect	Multiple components including diet and PA (Population-based)	Cost-consequences analysis (Health service delivery perspective)	Australian children aged 2 years modelled into adulthood	Lifetime	Australian dollars (2010)	3% discount rate on future costs and benefits	HALYs saved, life-years saved and health care cost savings	Assuming a reduction in BMI z-score of 0.13 in children aged 2 to 5 years, the scenario that assumed lifetime maintenance of effects resulted in 36,496 HALYs saved and health care cost savings of AUD 301 million over lifetime. Potentially cost-effective	22/24 (92%)
Canaway, A., et al. 2019 [105]	United Kingdom	Intervention schools: a multifaceted, 12-month, school-based intervention. Control schools: usual care	Diet and PA (School-based)	Cost-utility analysis (Public sector perspective)	Primary school children aged 6 to 7 years of mixed weight status	30 months: intervention was delivered over 12 months and follow-up was 18 months post-intervention	GBP (2014)	3.5% for costs and outcomes	Difference in BMI z-scores and QALY gained (CHU9D instrument)	For the base case, the ICER associated with the intervention was GBP 26,815 per QALY gained. Inconclusive due to uncertainty around effectiveness and varying WTP threshold of GBP 20,000–30,000 per QALY	21.5/24 (90%)
Kenney, E. L., et al. 2019 [83]	United States	Four drinking water interventions: Install water jet dispensers; Grab a Cup, Fill it Up; portable tap water dispensers;	Change in school drinking water environment (School-based)	Cost-benefit analysis (Societal perspective)	Students in kindergarten through eighth grade (K-8)	10-year time horizon	U.S. dollars (2015)	3% discount rate on future costs	Cost per case of childhood obesity prevented, cost per BMI unit reduced and health care cost savings per dollar invested	ICER not estimated. For the water jet intervention, the health care cost savings per dollar invested was USD 0.31 and the cost per BMI avoided was USD 105.29. Potentially cost-effective	21.5/24 (90%)

First Author and Year of Publication	Country	Intervention(s) and Comparator(s)	Intervention Component(s) and Target(s) (Setting)	Type of Economic Evaluation (Study Perspective)	Target Population and Age Range	Time Horizon/Follow-Up for Trials	Currency Unit (Price Year)	Discount Rate(s)	Measure(s) of Consequence(s)	Results and Cost-Effectiveness	CHEERS Quality Score (%)
		and install bottle-less water coolers.									
Sonntag, D., et al. 2019 [114]	Germany	Infant formula nutrition: lower protein (LP) versus higher protein (HP) intake groups	Diet: formula feeding (Multiple settings possible)	Cost-utility analysis (Societal perspective)	Infants born from uncomplicated singleton pregnancies	Lifetime	Euros (2015)	3% discount rate on future costs and benefits	QALY gained	Slightly higher QALYs and lower lifetime costs were estimated for LP relative to HP (negative ICER), therefore LP was deemed dominant and cost-effective	22.5/24 (94%)
Vieira, M. and Carvalho, G.S. 2019 [120]	Northern region, Portugal	The PHS-pro, a learning module promoting healthy choices versus a non-randomised control group	Water intake, TV viewing time, diet, and PA (School-based)	Cost-consequences analysis (Societal perspective)	Children 10 to 14 years old of varying weight status	10 months from baseline	Euros (2012)	NA	The per capita net cost of implementing the PHS-pro intervention in children versus cost of treating a case of adult obesity	The per capita net cost of implementing the intervention was 36.14 EUR/child/year compared to direct costs estimated for treating one obese adult in Portugal (3849.15 EUR/year). Inconclusive	14/24 (58%)
Bandurska, E., et al. 2020 [122]	Poland	12-month multidisciplinary obesity management comprising screening for overweight, specialist care, parental education, and specialist consultation versus no intervention	Diet, PA, and psychological support (Clinical and community based)	Cost-effectiveness analysis (Not stated/Provider perspective implied)	6- to 15-year-old overweight or obese children	7 years	PLN and Euros (not stated)	Not stated	Child removed from overweight or obesity	The costs of removing a child from the overweight group and obese group were PLN 27,758 (EUR 6463) and PLN 23,601 (EUR 5495), respectively. As noted earlier, the authors do not present any comparator costs or outcomes, so it is not clear if these estimates are incremental. Inconclusive	13/25 (52%)
Breheny, K., et al. 2020 [106]	United Kingdom	The Daily Mile: running or walking around school grounds for 15-min daily versus only the usual school health and wellbeing activities	PA (School-based)	Cost-utility analysis (Public sector perspective)	Children aged 4 to 11 years in all state-funded primary and junior schools	1 year	GBP (2017)	NA	QALY gained (CHU-9D)	The ICER for the Daily Mile intervention was GBP 7445 per QALY gained for the whole group and GBP 2492 per QALY gained for girls. Potentially cost-effective in girls, inconclusive for boys	22.5/24 (94%)

First Author and Year of Publication	Country	Intervention(s) and Comparator(s)	Intervention Component(s) and Target(s) (Setting)	Type of Economic Evaluation (Study Perspective)	Target Population and Age Range	Time Horizon/Follow-Up for Trials	Currency Unit (Price Year)	Discount Rate(s)	Measure(s) of Consequence(s)	Results and Cost-Effectiveness	CHEERS Quality Score (%)
Finster, M.P. and Feldman, J. 2020 [84]	United States	Onsite versus online support to implement a school-based wellness program	Diet and PA (School-based)	Cost-effectiveness analysis (Not stated/Societal perspective implied)	Students in low socioeconomic status neighbourhoods (age and weight status not specified)	4 years	U.S. dollars (not stated)	Not stated	Average percentage point increase in overall wellness (School Health Index) score	The cost per average percentage point increase in the overall wellness score was USD 336 and USD 256 for the onsite and online interventions, respectively. The onsite intervention potentially cost-effective	15/24 (63%)
Huse, O., et al. 2020 [98]	Australia	A national mandatory restriction on all price promotions for sugar-sweetened beverages (SSBs) available for purchase for take-home consumption in Australia versus no policy	Diet (Population-based, home-setting)	Cost-effectiveness analysis (Societal perspective)	The 2010 Australian population aged 2 to 100 years	Lifetime	Australian dollars (2010)	3% discount rate on future costs and benefits	HALYs gained	With a total HALYs gained estimated at 34,260 and the intervention cost an estimated AUD 17.0M, the policy was said to be dominant (cost-saving and health-promoting). Potentially cost-effective	22.5/24 (94%)
Oosterhoff, M., et al. 2020 [108]	The Netherlands	Two health promotion interventions in the school environment: Healthy Primary School of the Future (HPSF) and Physical Activity School (PAS) versus normal practice	Diet and PA (Primary school-based)	Cost-benefit analysis (Societal perspective)	Primary school children 4 to 12 years of age	2 years	Euros (2016)	Annual discount rate of 2.5% applied to investments and benefits	Outcomes across several sectors including health care, education, household, and leisure, were expressed in monetary terms and combined	Per child, investments of EUR 859 and EUR 1017 generated benefits of EUR 8 and EUR 49 for HPSF and PAS, respectively. Inconclusive	19.5/24 (81%)
Oosterhoff, M., et al. 2020 [109]	The Netherlands	Two health promotion interventions in the school environment: Healthy Primary School of the Future (HPSF) and Physical	Diet and PA (Primary school-based)	Cost-utility analysis (Societal perspective)	Primary school children 4 to 12 years of age	Lifetime	Euros (2018)	4% and 1.5% annual discount rates on future costs and benefits, respectively	QALY gained (meta-analysis)	From a societal perspective, PAS was dominated and HPSF was the most cost-effective with an ICER of EUR 19,734 per QALY gained. Potentially cost-effective	24/24 (100%)

First Author and Year of Publication	Country	Intervention(s) and Comparator(s)	Intervention Component(s) and Target(s) (Setting)	Type of Economic Evaluation (Study Perspective)	Target Population and Age Range	Time Horizon/Follow-Up for Trials	Currency Unit (Price Year)	Discount Rate(s)	Measure(s) of Consequence(s)	Results and Cost-Effectiveness	CHEERS Quality Score (%)
		Activity School (PAS) versus normal practice									
Tan, E. J., et al. 2020 [99]	Australia	POI sleep intervention alone and in combination with food, activity, and breastfeeding advice (FAB) versus usual care	Sleep modification, diet, PA, and breastfeeding (Home and clinical settings)	Cost-effectiveness and cost-utility analyses (Health funder perspective)	Healthy infants received the intervention up to the first 2 years of life and were then followed up to age 5 years	15 years	Australian dollars (2018)	Annual discount rate of 5% for costs and effects	QALY (meta-analysis) and unit BMI avoided	The ICER for the POI Sleep intervention alone was AUD 18,125 per QALY gained and was cost-effective at a willingness-to-pay threshold of AUD 50,000 per QALY	23/24 (96%)
Xu, H., et al. 2020 [61]	China	Three school-based childhood obesity interventions: NE intervention; PA intervention; and CNP intervention, which included the NE and PA interventions.	PA and diet (School-based)	Cost-utility and cost-benefit analyses (Societal perspective)	Baseline age of 10 years implied; childhood weight status not clearly stated. Parents, teachers, and health workers also received some of the interventions.	55 years (ages 10 to 65 years)	CNY and U.S. dollars (2019)	3% discount rate on future costs and benefits	QALYs and costs averted	The ICERs were CNY 10,335.2 (USD 1478.6) and CNY 4626.3 (USD 661.8) for CNP and NE respectively, compared with PA intervention. Estimates of monetary benefits were CNY 1.2, CNY 0.7, and CNY 0.4 per CNY 1 cost for CNP, NE, and PA interventions, respectively. CNP potentially cost-effective	17.5/24 (73%)

AASC: the active after-school communities programme; ACE: the assessing cost-effectiveness in obesity study; APPLE: A Pilot Programme for Lifestyle and Exercise; ARIC: Atherosclerosis Risk in Communities; BAEW: Be Active Eat Well; BMI: body mass index; CARDIA: Coronary Artery Disease Risk Development in Young Adults; CATCH[1]: Coordinated Approach to Child Health; CHOICES: Childhood Obesity Intervention Cost Effectiveness Study; CNP: comprehensive intervention; COCO: The Bristol Care of Childhood Obesity clinic; DALY: disability-adjusted life year; DKK: Danish crowns; DWCB: disordered weight control behaviours; ECE: early care and education; EDNP: energy-dense, nutrient-poor; EPOCH: Early Prevention of Obesity in Childhood; FBT: family-based treatment; FHC: food, health, and choices; GBP: pound sterling; GP: general practitioner; HALY: Health adjusted life year; ICER: incremental cost-effectiveness ratio; ITT: intention-to-treat; LEAP: the live, eat and play trial; LYG: life year gained; MCG FitKid: the Medical College of Georgia FitKid Project; MEPS: Medical Expenditures Panel Survey; MET: metabolic equivalent; MPA: moderate physical activity; MVPA: moderate to vigorous physical activity; NA: not applicable; NE: nutrition education intervention; NHANES: National Health and Nutrition Examination Survey; NHF: National Heart Forum; P&C: parent and child; PA: physical activity; PA4E1: Physical Activity 4 Everyone; PC-COCO: Primary Care-Care of Childhood Obesity; PE: physical education; PHS-pro: Planning Health in School; PIF: potential impact fraction; PLN: Polish zloty; POI: Prevention of Overweight in Infancy; PSA: probabilistic sensitivity analysis; QALYs: quality-adjusted life years; RCT: randomised controlled trial; CNY: Chinese yuan renminbi; SPARK: The Sports, Play, and Active Recreation for Kids trial; SSB: sugar-sweetened beverages; TV AD: television advertisements; URMEL-ICE: Ulm Research on Metabolism Exercise and Lifestyle Intervention in Children; WC: waist circumference; WHtR: waist-to-height ratio; WSB: Walking School Bus programme; WTP: willingness to pay.

Table S6. Descriptive analysis of the key assessment items for studies on the impact of childhood obesity on human capital.

First Author and Year of Publication	Country	Type of Study/Dataset	Period Considered	Target Population	Sample Size	Stratification	Exposure	Outcome(s) Estimated	Main Results	Conclusion
Sabia, J. J. 2007 [140]	United States	Individual-level longitudinal/Add Health	1994–1995 (wave 1) to 1995–1996 (wave 2)	A nationally representative sample of 14- to 17-year-olds	5129 adolescents	Sex and race (White, non-White)	Overweight and obesity (BMI z-score and pounds)	CP (High school GPA)	A difference in weight of 50 to 60 pounds (approximately two standard deviations) was associated with a 0.2-point difference in GPA for white the average white female	Significant negative relationship for white females and non-white males, but not for non-white females or white males
Ding, W., et al. 2009 [141]	United States	Individual-level longitudinal/GATOR	1999 to 2003	Adolescents in grades 10, 11, and 12	893 adolescents	Sex	Obesity (BMI)	CP (High school GPA)	Obesity led to a 0.45-point decrease on GPA for the full sample. For females and males, 0.5-point decrease and 0.1-point decrease were estimated, respectively	Substantial impacts on the CP of female adolescents, but males were not significantly impacted
Kaestner, R. and M. Grossman 2009 [142]	United States	Individual-level longitudinal/NLSY97	1986 to 2004	Children between the ages of 7 and 12 years	2200 observations per two-year age group	Sex and age	Overweight and obesity (BMI z-score)	CP (PAIT: maths and reading) and EA (Grade attainment)	The study found that overweight or obese children achieved similar test scores and grade attainment as children with average weight	Non-statistically significant estimates
Okunade, A. A., et al. 2009 [157]	United States	Individual-level longitudinal/Add Health	1994–1995 (wave 1) to 2001–2002 (wave 3)	Students in grades 7 to 12 (aged 12 to 17 years)	8388 individuals	Sex and race (White, Black, Hispanic, Asian)	Overweight and obesity (BMI z-score)	EA (on-time high school graduation)	Asian females were most affected, with lower probabilities of on-time graduation of 38% and 14%, respectively, for obese or overweight subgroups. In white females, a strong negative effect of −9% was estimated for overweight but not for obesity. No significant effect of excess weight on academic attainment was found for males of any race or African Americans	Statistically significant negative relationship with on-time high school completion for females. Race: adverse effects primarily to white and Asian females but no significant effects for African Americans
Averett, S. L. and D. C. Stifel 2010 [143]	United States	Individual-level longitudinal/NLSY97	1979 to 2002	The children ages 6 to 13 years	20,856 child years	Sex and race (White, Black)	Overweight (BMI z-score)	CP (PIAT: math and reading)	The study found that overweight white boys had math and reading scores approximately 1 SD below the	Statistically significant negative relationship for CP in white and black children of both sexes

First Author and Year of Publication	Country	Type of Study/Dataset	Period Considered	Target Population	Sample Size	Stratification	Exposure	Outcome(s) Estimated	Main Results	Conclusion
									mean, while overweight white girls had lower math scores. Overweight black boys and girls were estimated to have lower reading scores	
Fletcher, J. M. and S. F. Lehrer 2011 [144]	United States	Individual-level longitudinal/Add Health	1994–1995 (wave 1) to 2001–2002 (wave 3)	Twins and siblings, mean age 17 years	1684 children comprising twins and siblings	Sex and race (White, African American, Hispanic, Asian)	Overweight and obesity (BMI z-score and pounds)	CP (High school GPA and PIAT: verbal)	The study estimated that being overweight was associated with a small decrease in academic performance that was not statistically significant at the 5% level	Non-statistically significant estimates
Chen, L. J., et al. 2012 [145]	Taiwan	Individual-level longitudinal/Taichung City primary district	2002 to 2008	First-grade students in Taichung City	409 children	Sex	Overweight and obesity (BMI z-score)	CP (mean scores in language, maths, science, history)	The study found no significant relationship between initial obesity or change in weight status at grade 1 and academic performance at grade 6	Non-statistically significant estimates
Scholder, S. v. H. K., et al. 2012 [146]	United Kingdom	Individual-level longitudinal/ALSPAC	3-year period (ages 9 to 11 and 11 to 14 years)	Children aged 9 years and 11 years	3001 children	Race (White only)	Fat mass (measured by DXA scan)	CP (exam result on the Key Stage 3 (KS3) test)	The study concluded that body weight status was unlikely to be causally related to academic achievement in adolescence	Non-statistically significant estimates
Bisset, S., et al. 2013 [147]	Canada	Individual-level longitudinal/QLSCD	1 year of follow-up at average age 8.2 years of age	Children aged 4 to 7 years	1959 children	None	Overweight (BMI z-score)	CP (KABC: average of reading, writing, maths)	The study found that children within the lowest BMI trajectory had a reduced reading comprehension (KABC-pt2) of 3.81 points (p = 0.042) compared with their normal-weight counterparts, but children with stable overweight were not at higher risk for poorer academic or cognitive outcomes	Non-statistically significant estimates for excess weight compared with normal weight
Capogrossi, K. and W. You 2013 [148]	United States	Individual-level longitudinal/ECLS-K	1998–1999 school year to	A nationally representative	21,260 children	Sex, race, and school grade (White, minority)	Underweight, overweight and	CP (ECLS-K: math and reading IRT scores)	The study IV QR estimates showed that weight positively affected white students with	Mixed relationship: larger significant impact on lower-performing

First Author and Year of Publication	Country	Type of Study/Dataset	Period Considered	Target Population	Sample Size	Stratification	Exposure	Outcome(s) Estimated	Main Results	Conclusion
			2006–2007 school year	sample of kindergartners			obesity (BMI z-score)		emphasis on the lower end of the academic performance distribution for 8th-grade math and reading scores where magnitudes ranged from an increase in scores of 1.04 points to 2.80 points	students; for minority students, either a negative or nonsignificant impact
Lu, Y.-L., et al. 2014 [149]	Taiwan	Individual-level longitudinal/TEPS	2001 (7th grade) to 2003 (9th grade)	A nationally representative sample of children in the 7th grade	8690 students	Sex	Underweight and overweight (BMI)	CP (CCA scores)	The study estimated a statistically significant relationship between weight and test scores using PSM method, but not with OLS. The PSM models estimated an impact of weight status 3 to 8 times larger than that estimated by OLS	Negative relationship for academic performance in both male and female students
Amis, J. M., et al. 2014 [20]	United States	Individual-level longitudinal/Add Health	1994 to 1995 and followed over 13 years	A nationally representative sample of students with mean age of 16	11,308 students	Sex and race (White, Blacks, and Hispanics)	Obesity (BMI z-score)	EA and LMO (adult earnings/income)	The study found that obese adolescents who went on to attend college were about 9% less likely to graduate compared to their nonobese peers and earned 7.5% less as adults. Obese females and whites were about 12% less likely to complete a college degree than nonobese adolescents	No significant impact on high school graduation but significant effects for college graduation and future income among blacks and females
Lundborg, P., et al. 2014 [21]	Sweden (and separate analysis using U.K. and U.S. cohorts)	Individual-level longitudinal/the SNSA register of enlistment	1984 to 2003	Male siblings who enlisted for the military at age 18 years and lived in Sweden in 1999	145,193 teenage siblings	Sex (male)	Overweight and obesity (BMI)	LMO (adult earnings/income)	For the Swedish cohort, in comparison with teenagers of normal weight, overweight and obese adolescents earned 6% and 18% less, respectively, between ages 28 and 38 years. In the U.K. cohort, the study estimated that being obese at 16 was associated with 38% lower earnings at age 42.	Substantial penalties for excess weight in teenage years with respect to adult earnings, both in Sweden and in cohorts from the United States and the United Kingdom

First Author and Year of Publication	Country	Type of Study/Dataset	Period Considered	Target Population	Sample Size	Stratification	Exposure	Outcome(s) Estimated	Main Results	Conclusion
									Using NLSY79 data, the study estimated that men who were obese at ages 16 to 24 were likely to earn 18% lower at ages 39 to 42, relative to their normal-weight counterparts	
Afzal, A. S. and S. Gortmaker 2015 [150]	United States	Individual-level longitudinal/NLSY97	Two cohorts: 1988–1994 and 1994–2000	Children aged 2 through 8	Cohort 1, *n* = 2672 and Cohort 2, *n* = 1991	Sex	Always obese, became obese/non-obese (BMI z-score)	CP (PPVT-R, PIAT Math, PIAT Reading and WISC-R)	The study concluded that childhood obesity was unlikely to be causally related to cognitive performance.	Non-statistically significant estimates
Black, N., et al. 2105 [151]	Australia	Individual-level longitudinal/LSAC	2004 to 2012 (5 waves)	Children aged 4 to 5 years in the first wave (children in Cohort K)	4983 children	Sex	Obesity (BMI z-score)	CP (NAPLAN: maths and reading)	The study found that obesity was negatively related to cognitive achievement for boys but not girls. The IV estimate for literacy indicated that obese boys performed 1.3 SDs worse than normal-weight boys.	Significant negative effect for boys but non-statistically significant estimates for girls
Kenney, E., et al. 2015 [152]	United States	Individual-level longitudinal/ECLS-K	1998–1999 to 2006–2007 school years	A nationally representative sample of children in the 5th grade	3362 children	Sex	Overweight and obesity (BMI z-score)	CP (ECLS-K: math and reading IRT scores)	The study reported significantly worsening teacher perceptions associated with an increase in BMI z-score from the fifth to eighth grade. However, the study did not establish an independent association between change in BMI z-score and change in objectively measured CP over the same period	Non-statistically significant estimates
Kranjac, A. W. 2015 [153]	United States	Individual-level longitudinal/ECLS-K	1998–1999 to 2006–2007 school years	A nationally representative sample of kindergartners	5034 children	None	Overweight and obesity (BMI z-score and BMI)	CP (ECLS-K: math) and self-efficacy	The study found that the math trajectories of overweight children with high levels of self-efficacy exceeded those of trajectories of overweight children with high levels of self-efficacy by	Self-efficacy was associated with math outcomes in overweight, but not obese children

First Author and Year of Publication	Country	Type of Study/Dataset	Period Considered	Target Population	Sample Size	Stratification	Exposure	Outcome(s) Estimated	Main Results	Conclusion
									3.62 points ($p < 0.005$). Estimates were not statistically significant in obese children	
Sabia, J. J. and D. I. Rees 2015 [154]	United States	Individual-level longitudinal/Add Health	1994–1995 (wave 1) to 2007–2008 (wave 4)	A nationally representative sample of adolescents (14 years)	20,028 adolescents	Sex	Overweight and obesity (BMI z-score and pounds)	CP (High school GPA) and EA (High school diploma and college completion)	The study's TSLS estimated that having excess weight was associated with a 0.341-point reduction and a 0.252 reduction in the probability of college completion in GPA for females but less significant for males	Negative relationship with female academic achievement and less significant in boys
Von Hinke, S., et al. 2016 [155]	United Kingdom	Individual-level longitudinal/ALSPAC	3-year-period (11 to 14 years)	Children aged 11 years	4844 children	Sex and race (White only)	Fat mass (measured by DXA scan)	CP (exam result on the Key Stage 3 (KS3) test)	The study found that using OLS regression, leaner children recorded better CP than fatter children. In the study's genetic IV analysis; however, there was no evidence that CP was driven by children's fat mass	Non-statistically significant estimates
Pinkston, J. C. 2017 [158]	United States	Individual-level longitudinal/NLSY97	1997 to 2009	Children aged 12 to 16 years in 1996	1473 white men and 1060 white women	Sex and race (White only)	Overweight, obesity and severe obesity (BMI)	LMO (hourly wage)	The study estimated a penalty of about 17% for men who were severely obese in the previous year. An initial penalty of 13% was estimated for women entering the labour market with a BMI over 37.	A childhood history of excess weight had a significantly large negative effect on wages
Shi, H. and C. Li 2018 [156]	Australia	Individual-level longitudinal/LSAC	2004 to 2014 (6 waves)	Children aged 4 to 5 years in the first wave	10,858 observations	Grade level (Year 3, Year 5, Year 7, and Year 9)	Overweight and obesity (BMI z-core)	CP (NAPLAN: reading, grammar, spelling, writing, and numeracy)	The IV model estimated that obesity was associated with 0.80 SD, 1 SD, 0.84 SD, 0.91 SD, and 0.98 SD below the mean for reading, grammar, spelling, writing, and numeracy, respectively, when using only the biological	Negative impact on academic performance with a larger negative impact for senior year students, particularly on numeracy

First Author and Year of Publication	Country	Type of Study/Dataset	Period Considered	Target Population	Sample Size	Stratification	Exposure	Outcome(s) Estimated	Main Results	Conclusion
									mother's BMI as the instrument	

Add Health: National Longitudinal Study of Adolescent Health; ALSPAC: U.K. Avon Longitudinal Study of Parents and Children; CCA: comprehensive cognitive ability; CDC: Centres for Disease Control and Prevention; CP: cognitive performance; DXA: dual-energy X-ray absorptiometry; EA: educational attainment; ECLS-K: Early Childhood Longitudinal Study-Kindergarten Class Assessment; GATOR: Georgetown Adolescent Tobacco Research GATOR study; GPA: grade point average; IOTF: International Obesity Task Force growth charts; IRT: Item Response Theory; KABC: Kaufman's Assessment Battery for Children; LMO: labour market outcomes; MCS: Millennium Cohort Study; NAPLAN: National Assessment Program—Literacy and Numeracy; NCDS: National Child Development Study; NLSY97: U.S. National Longitudinal Survey of Youth 1997; PIAT: Peabody Individual Achievement Test; PPVT-R: Peabody Picture Vocabulary Test-Revised; PSM: propensity score matching; QLSCD: Quebec Longitudinal Study of Child Development; SNSA: Swedish National Service Administration; TEPS: Taiwan Education Panel Survey data; WISC-R: Wechsler Intelligence Scale for Children-Revise.
